# The effectiveness of intervention in hepatitis C patients and improvement in their referral rate

**DOI:** 10.1080/07853890.2024.2346537

**Published:** 2024-05-02

**Authors:** Jiao-Qian Ying, Yu-jiao Zhang, Yun-qing Xia, Hao-di Ma, Tie Zhang, Ling Tu, Ai-hui Zheng, Peng Gao, Wei-hua Wang

**Affiliations:** a Department of Medical Affairs, China-Japan Friendship Hospital, Beijing, China; b Department of Infectious Disease, China-Japan Friendship Hospital, Beijing, China; c School of Mathematics and Statistics, Beijing Institute of Technology, Beijing, China; d Department of Computer & Information Science & Engineering, University of Florida, Gainesville, FL, USA; e Department of Clinical Laboratory, China-Japan Friendship Hospital, Beijing, China; f Department of IT, China-Japan Friendship Hospital, Beijing, China; g Department of Nosocomial Infection Control and the Center for Disease Control and Prevention, China-Japan Friendship Hospital, Beijing, China

**Keywords:** Critical value, effectiveness validation, patients with hepatitis C, referral rate, SMS

## Abstract

**Background:**

To investigate the effectiveness of the intervention with critical value management and push short messaging service (SMS), and to determine improvement in the referral rate of patients with positive hepatitis C antibody (anti-HCV).

**Methods:**

No intervention was done for patients with positive anti-HCV screening results from 1 January 2015 to 31 October 2021. Patients with positive anti-HCV results at our hospital from 1 November 2021 to 31 July 2022 were informed vide critical value management and push SMS. For inpatients, a competent physician was requested to liaise with the infectious disease physician for consultation, and patients seen in the OPD (outpatient department) were asked to visit the liver disease clinic. The Chi-square correlation test, one-sided two-ratio test and linear regression were used to test the relationship between intervention and referral rate.

**Results:**

A total of 638,308 cases were tested for anti-hepatitis C virus (HCV) in our hospital and 5983 of them were positive. 51.8% of the referred patients were aged 18–59 years and 10.8% were aged ≥75 years. The result of Chi-square correlation test between intervention and referral was *p* = .0000, *p* < .05. One-sided two-ratio test was performed for statistics of pre-intervention referral rate (*p*1) and post-intervention referral rate (*p*2). Normal approximation and Fisher’s exact test for the results obtained were 0.000, *p* < .05, and the alternative hypothesis *p*1 − *p*_2_ < 0 was accepted. The linear regression equation was referral = 0.1396 × intervention + 0.3743, and the result model *p* = 8.79e − 09, *p* < .05. The model was significant, and the coefficient of intervention was 0.1396.

**Conclusions:**

The interventions of critical value management and push SMS were correlated with the referral rate of patients with positive anti-HCV.

## 1. Background

Hepatitis C is a major cause of chronic liver disease. In 2015, of the 71 million people with hepatitis C virus (HCV) infection globally [1], only 20% were diagnosed and only 7% had received treatment [2]. Furthermore, global HCV elimination is dependent on proportions of people diagnosed and treated in high-burden countries including China, Egypt, India and Pakistan [3], yet less than 25% of those infected have been diagnosed [4]. With the use and approval of direct-acting antiviral (DAA) agent regimens in China, patients with hepatitis C can achieve more than 90% sustained virological response (SVR) with DAA therapy [5]. Early detection and successful referral are a critical requirement for the elimination of hepatitis C in China. Currently, the false negative rate of hepatitis C in China is high with low referral and treatment rates, and the awareness levels amongst healthcare staff for the necessity of screening and treatment of hepatitis C are low. Even though patients with positive hepatitis C antibody (anti-HCV) were identified during in-hospital screening, the percentage of patients visiting the infection department for consultation or referral was low [6]. Based on the current situation of low awareness rate and high cure rate, it is particularly important to improve patients’ awareness rate and increase their referral rate. This study effectively improved the referral rate of patients by means of short messaging service (SMS), push of critical value and prompt of positive results in examination report. On comparison of data between the two groups before and after the intervention, this study revealed that the intervention has a positive effect on the improvement of the referral rate of patients with positive anti-HCV and provides a theoretical basis for improving the referral rate of patients with hepatitis C in hospitals.

## 2. Objects and methods

### 2.1. Study objects

This is a single-centre, retrospective, cohort-controlled study. All patients who visited and underwent anti-HCV test from 1 January 2015 to 31 July 2022 in China-Japan Friendship Hospital were mainly included. The name, gender, age, cell phone number, anti-HCV results and hepatitis C virus ribonucleic acid (HCV-RNA) results of the patients were collected in the Hospital Laboratory Information System (LIS). Our screening condition is that patients with positive anti-HCV results and no HCV-RNA examination in our hospital will be intervened. In the hospital for surgery, anti-HCV screening will be done by physical examination before invasive operation. Patients who have already been referred to a specialty are not the subjects of our intervention. The purpose of our intervention is to successfully refer non-specialist antibody positive patients to the specialist (infectious disease unit) for further treatment.

Inclusion criteria: (1) patients who visited and underwent anti-HCV test at China-Japan Friendship Hospital from 1 January 2015 to 31 July 2022; (2) with positive anti-HCV result; (3) no missing information to be collected.

Exclusion criteria: (1) specimens sent from other hospitals; (2) repeated tests with the same test report results; (3) any missing data.

This study was conducted with approval from the Ethics Committee of China-Japan Friendship Hospital (2020YFC2004803). This study was conducted in accordance with the declaration of Helsinki. Written informed consent was obtained from all participants.

### 2.2. Test method

#### 2.2.1. Reagent

Hepatitis C antibody kit (Shenzhen YHLO Biotechnology Co., Ltd., Shenzhen, China, stored at 2–8 °C); instrument: YHLO Full-Automatic Chemiluminescence Instrument iFLASH3000 was used for the test.

#### 2.2.2. Reagents

Hepatitis C Virus Nucleic Acid Quantitative Test Kit (produced by Sun Yat-sen University Daan Gene Co., Ltd., Guangzhou, China, stored at −20 °C) and nucleic acid extraction or purification reagents (produced by Sun Yat-sen University Daan Gene Co., Ltd., Guangzhou, China, stored at room temperature); instrument: Daan smart32 nucleic acid extractor was used for nucleic acid extraction and Roche COBAS480 nucleic acid detection system was used for nucleic acid amplification and detection.

### 2.3. Study method

#### 2.3.1. Interventions

There are two ways for outpatients: the first one is, patients who were positive for anti-HCV test were informed by critical value management and push SMS from 1 November 2021. Patients who were positive for anti-HCV in the outpatient clinic were informed through the hospital SMS platform to the cell phone number provided by the patient with the following content ‘Hello, your hepatitis C antibody test result at China-Japan Friendship Hospital is abnormal, we suggest you visit the "liver disease clinic" for further examination and treatment. [China-Japan Friendship Hospital 84205976].’ The second one is, mark the positive result ‘↑’ in the test report form, and tell the patient that ‘hepatitis C antibody results are abnormal, it is recommended that you go to the infectious diseases department for further examination.’

The information of inpatients with positive anti-HCV was first sent to the competent physician through a pop-up window in the hospital information system (HIS) system of the hospital, and a text message was sent to the competent physician of the patient in the SMS platform, prompting the physician to further check the HCV-RNA of the patient, and if the result of HCV-RNA was positive, a consultation with infectious disease physician was required. The professional staff of the medical office will track the HCV-RNA test results of anti-HCV patients, and if they are positive, check whether the doctor in charge invites the infectious diseases doctor to consult with the patient. If the consultation invitation is not initiated, call the doctor to remind the doctor to issue the consultation application. The closed-loop management mode of screening, informing, monitoring and feedback is formed to ensure successful referral of patients and improve referral rate.

The data from 1 January 2015 to 30 October 2021 were not intervened by critical value management and push SMS, and the data from 1 November 2021 to 31 July 2022 were intervened.

#### 2.3.2. Relevant concepts

##### 2.3.2.1. Patient referral

Outpatients were treated promptly at the Infectious Disease Department or the Hepatology Department, and inpatients were promptly consulted by the Infectious Disease Department or the Hepatology Department and referred to the Infectious Disease Department or the Hepatology Department in due course [6].

##### 2.3.2.2. Consultation rate in specialty or attendance rate (referral rate)

Number of anti-HCV-positive patients in specialty or consultation/number of anti-HCV-positive patients in the same period × 100% [7].

### 2.4. Statistical analysis

#### 2.4.1. Main study indicators

Gender, age, intervened or not, referred or not, diagnosed or not, and turned to negative or not.

#### 2.4.2. Data processing

The age was uniformly converted to years, and those less than one month were recorded as 0 years. Any case containing one or more of the main study indicators that was missing was excluded from the statistical analysis.

#### 2.4.3. Statistical methods

Levene’s test and two-sample *t*-test were used for inter-group comparisons of numerical variables. Chi-square test was performed for the subtype variables to analyse their correlation. The one-sided two-ratio test (also known as the one-sided two-sample *z* test) [8] was used to compare the differences in referral rates between groups. The linear regression model was used to further verify the correlation between the variables and the magnitude of the effect. The test criterion *α* = 0.05.

#### 2.4.4. Analytical tool

Microsoft Office Excel 2017 (Redmond, WA) was used for data collection and summarization. Data statistics were performed using Jupyter Notebook and Minitab 21.1 (Obtained a copyright license) based on the Python 3.10 environment.

## 3. Results

**Figure 1.**
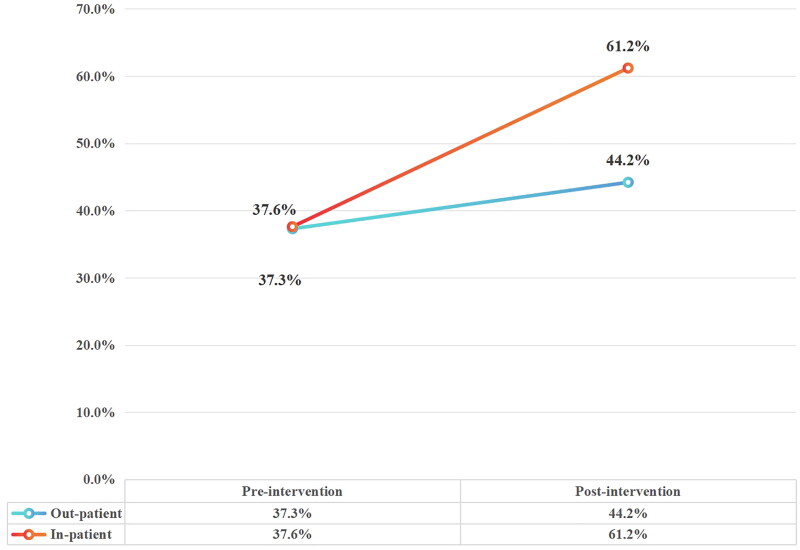
The specific data of 638,308 cases were tested for anti-HCV in our hospital.

A total of 638,308 cases were tested for anti-HCV in our hospital from 2015 to 31 July 2022, and 5983 of them were positive. The specific data are shown in Table 1 and Figure 1.

**Table 1.** Basic data.

Items		Pre-intervention	Post-intervention
Anti-HCV	Negative	556,880	81,428
	Positive	5552	431
Anti-HCV (+)	Outpatient	2926	248
	Inpatient	2626	183
Referral rate	Outpatient	37.30%	44.20%
	Inpatient	37.60%	61.20%
Gender	Male	2588	210
	Female	2954	221
Referred		2078	221
Referral patient HCV-RNA (+) person-time (proportion)		447 (21.5%)	46 (20.8%)
Not referred		3474	210
Reported		40	40

Patients were divided into pre-intervention and post-intervention groups. Levene’s test was performed to study the homogeneity of variances in both groups, and Levene’s result (statistic = 2.7663, *p* value = .0963) was obtained. Since the *p* value was greater than .05, it indicated that there was no significant difference in the variance of age between both groups. The two-sample *t*-test was then performed for all ages under the homogeneity of variances and the resultant T test_indResult (statistic = 1.6956, *p* value = .0900) was obtained. Since the *p* value was greater than .05, it indicated that there was no significant difference in age between the patients in the pre-intervention and post-intervention groups. 51.8% of the referred patients were aged 18–59 years and 10.8% were aged ≥75 years.

**Figure 2.**
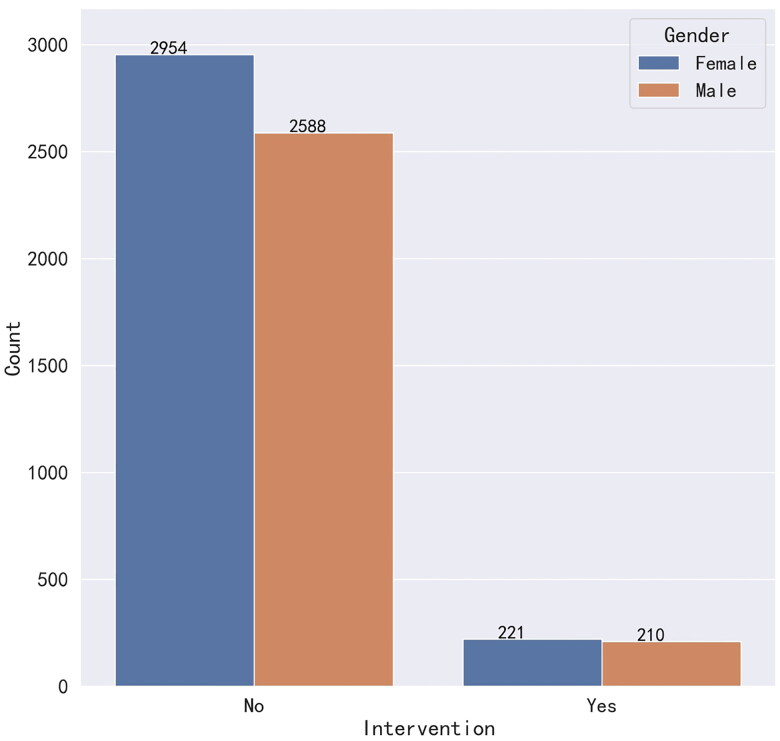
Gender association with the intervention.

The patients were divided into pre-intervention and post-intervention groups to test whether gender was associated with the intervention, and graphs were drawn with or without the intervention, as shown in Figure 2, which shows that the distribution of males and females in the pre-intervention and post-intervention groups was similar. The exact result was then obtained with the Chi-square correlation test, and the Chi-square value = 0.517 and *p* value = .4710. Since the *p* value was greater than .05, it indicated that the gender of the enrolled patients did not correlate with the presence or absence of the intervention.

The patients were divided into pre-intervention and post-intervention groups, and a Chi-square correlation test was performed between the intervention and referral groups, and the result obtained was *p* value = .0000. Since *p* value was less than .05, it indicated that there was a correlation between the intervention and referral.

The referral rate was 0.3743 for the pre-intervention group and 0.5139 for the post-intervention group. The referral rate improved significantly after the intervention, which indicated that the intervention had a significant promotion effect on referrals (see Table 2).

**Table 2.** Referral is correlated with intervention using Chi-square test.

	Pre-intervention	Post-intervention	Total
Not referred	3474	210	3684
	3418.0	266	
Referred	2078	222	2300
	2134.0	166.02	
Missed	69	4	^a^
Chi-square test			
Pearson = 33.015; *p* value = .000; *p* < .05			
Likelihood ratio = 32.127; *p* value = .000; *p* < .05			

^a^ The missed column is the amount of data with missing information in the collected basic data, which is not summarized and meaningless.

To accurately test the referral rate difference between the pre-intervention and post-intervention groups, the groups were statistically tested with one-sided two-ratio test, and the results showed that the normal approximation and Fisher’s exact test were both 0.000, *p* < .05, and the alternative hypothesis *p*1 − *p*_2_ < 0 was accepted. Based on the data in Table 3, the referral rate in the post-intervention group was significantly higher than that in the pre-intervention group and supported the fact that effective intervention was helpful in increasing the referral rate of patients with positive anti-HCV (see Supplemental Materials).

**Table 3.** Two-ratio test of pre-intervention and post-intervention referral rates.

	*N*	Event	Sample *p*
Pre-intervention	5552	2078	0.374
Post-intervention	432	222	0.514
*Method*			
*p*_1_: pre-intervention referral rate			
*p*_2_: post-intervention referral rate			
Difference = *p*_1_ − *p*_2_			
*Test*			
Original hypothesis: H_0_: *p*_1_ − *p*_2_ = 0			
Alternative hypotheses: H_1_: *p*_1_ − *p*_2_ < 0			
Method		*Z*	*p*
Normal approximation		−5.60	.000
Fisher’s exact test			.000
*p* < .05			

## 4. Discussion

The current World Health Organization (WHO) goal for hepatitis C is to have 30% of infected patients aware of their infection status by 2020, with 50% of eligible patients receiving treatment services and achieving SVR [9]. There are about 10 million patients with chronic hepatitis C in China, accounting for 14% of the total number of patients worldwide, while about 2.5 million patients are in urgent need of antiviral therapy and are not even aware of their infection status [10]. The low referral rate of hepatitis C in China may be due to multiple reasons, such as lack of knowledge about hepatitis C and positive anti-HCV. Hepatitis C is not taken seriously by patients, and they believe that no treatment is required if there are no symptoms, or that it cannot be cured. The finer classification of departments in the hospital makes it impossible for patients to find appropriate departments to visit; and it is not taken seriously by clinicians as well, especially those who are not specialized in infection management. Micro-elimination is a pragmatic approach that breaks national elimination goals into smaller goals for subpopulation groups and enables the delivery of treatment and prevention interventions more quickly and efficiently [11]. Based on the above studies, anti-HCV screening for patients who visit hospitals for invasive procedures such as surgery and endoscopy makes it easier to target positive populations, and allows patients to receive early standardized treatment, as long as an effective approach is taken to enable them to be successfully referred. To improve the referral rate of hepatitis C, our hospital adopted push SMS to inform outpatients with positive anti-HCV to seek further medical treatment in a specific department, and left their phone numbers in the SMS to facilitate further consultation for patients. This allows patients to have clearer information about the departments and consultation pathways, thus improving patient compliance in follow-up consultation and enabling them to receive continuous medical services, which can then improve the treatment rate. For inpatients with positive anti-HCV, patient information is first pushed to clinically competent physicians through the HIS system in the form of critical value, so that physicians can receive the relevant information prompting them to conduct HCV-RNA and other related tests for the patients on time, and also permitting the treatment status to be tracked continuously. Second, SMS messages are sent to the competent physician of the patient with positive anti-HCV, so they can be informed of the patient’s test results in a timely manner, which is a very practical solution. The medical office has set up a ‘hepatitis C special’ work, which is undertaken by special personnel. I was responsible for organizing multi-departments of the Information Department, Infectious Diseases Department and clinical laboratory to coordinate, designing the procedures of patient screening, diagnosis, referral, treatment and follow-up, supervising the implementation, and responsible for data collection, statistics, summary and feedback, which also promoted the improvement of referral rate. In addition, propaganda materials related to hepatitis C are placed in dental clinics and physical examination centres to guide patients to take the initiative to carry out hepatitis C screening, so that patients can be early detection, early referral and early treatment. The referral rate for patients with positive antibodies was 37% before the intervention and 51% after the intervention, the rate of outpatient referral ranged from 37.3% to 44.2%, and the rate of inpatient referral ranged from 37.6% to 61.2%. At the same time, the medical department has the person in charge of the hepatitis C special work, who will collect the test results of anti-HCV and HCV-RNA through the LIS system every day, and check whether the clinicians have issued the HCV-RNA test to the patients with anti-HCV positive, and whether they have issued the medical advice of referral/consultation to the patients with HCV-RNA positive through HIS system. Supervise clinical referrals, which indicated that our approach was very effective. The ‘small scale clearance’ strategy in hospitals [11,12] may promote necessary referral behaviour of physicians through the critical value and push SMS, to inform patients with positive anti-HCV about the meaning and pathway of referral, thus increasing the referral rate and reducing the false negative rate. Our method is simple and easy to implement, and is worthy of being replicated in hospitals. In addition, the reporting rate of hepatitis C was 9% before the intervention and 89% after the intervention, which indicated that the inclusion of positive anti-HCV in the hospital’s critical value management was helpful in improving physicians’ awareness of hepatitis C and had a positive effect on improving patient referrals.

There are still shortcomings in this study: the referral rate of outpatients was not significantly improved by intervention, because we did not follow-up by phone after the text message notification. Perhaps this notification method did not attract patients’ attention and they did not see a doctor, or maybe the patients went to other hospitals and we did not include the referred patients. Such speculation comes from the data in our hospital showing that some patients were not initially screened in our hospital, they directly went to our hospital for further detection of HCV-RNA, after diagnosis for treatment. We will further improve outpatient interventions, such as telephone follow-up, to increase outpatient referral rates. This is single-centre study; the duration of the intervention is short and data are inadequate. It is a retrospective, non-randomized controlled study. Because our work focused more on the successful referral of anti-HCV positive patients to the liver disease clinic, and standardized antiviral treatment as early as possible, and did not collect the information of patients’ underlying diseases (such as the existence of liver damage?).

## 5. Conclusions

In summary, with various DAAs receiving approval in China and the inclusion of some drugs under medical insurance, the cost for eradicating hepatitis C has gradually decreased, and there is an unprecedented opportunity to eliminate hepatitis C. However, due to the hidden onset and long duration of hepatitis C, most patients have no obvious symptoms, and many HCV carriers are not detected [13]. Therefore, this simple and effective intervention enables successful referral for early diagnosis and early standardized antiviral treatment, and contributes to the global strategy to ‘eliminate viral hepatitis by 2030’ [14].

## Supplementary Material

Supplemental Material

## Data Availability

The datasets used and/or analysed during the current study are available from the corresponding author on reasonable request.
