# Supplementary material for: The effectiveness of intervention in hepatitis C patients and improvement in their referral rate
Source: Ann Med. 2024 May 2;56(1):2346537. doi: 10.1080/07853890.2024.2346537 (PMC11067552; doi:10.1080/07853890.2024.2346537)
Supplement: Supplemental Material [file IANN_A_2346537_SM3370.docx]

Linear regression with intervention as the independent variable and referral as the dependent variable resulted in the p value = 8.79e-09 for the outcome model.

The model was significant at p < 0.05, with a coefficient of 0.1396 for the intervention, which indicated that the intervention was positively associated with referral and re-supported that the intervention could be helpful in increasing the referral rate of patients with positive anti-HCV. The linear regression equation was Referral = 0.1396*intervention + 0.3743. See Tables 1, 2, and 3.

Table 1 Linear regression analysis of interventions and referrals

| Dep.Variable: | Referred | R-squared: | 0.006 |
| --- | --- | --- | --- |
| Model: | OLS | Adj.R-squared: | 0.005 |
| Method: | Least Squares | F-statistic: | 33.19 |
| Date: | Thu,25 Aug 2022 | Prob(F-Statistic): | 8.79e-09 |
| Time: | 18:50:40 | Log-Likelihood: | -4162.1 |
| No.Observations: | 5984 | AIC: | 8328. |
| Df Residuats: | 5892 | BIC: | 8342. |
| Df Model: | 1 | | |
| Covariance Type | nonrobust | | |

Table 2: Linear regression parameter results

|  | Coef | Std err | t | P>[t] | [0.025 | 0.975] |
| --- | --- | --- | --- | --- | --- | --- |
| Intervened | 0.1396 | 0.024 | 5.761 | 0.000 | 0.092 | 0.187 |
| Intercept | 0.3743 | 0.007 | 57.480 | 0.000 | 0.362 | 0.387 |

Table 3: Linear regression hypothesis test

| Omnibus: | 25057.712 | Durbin-Watson: | 1.993 |
| --- | --- | --- | --- |
| Prob(Omnibus): | 0.000 | Jarque-Bera(JB): | 989.070 |
| Skew: | 0.474 | Prob(JB): | 1.68e-215 |
| Kurtosis: | 1.249 | Cond.No. | 3.89 |
